# Transected Tendon Treated with a New Fibrin Sealant Alone or Associated with Adipose-Derived Stem Cells

**DOI:** 10.3390/cells8010056

**Published:** 2019-01-16

**Authors:** Katleen Frauz, Luis Felipe R. Teodoro, Giane Daniela Carneiro, Fernanda Cristina da Veiga, Danilo Lopes Ferrucci, André Luis Bombeiro, Priscyla Waleska Simões, Lúcia Elvira Alvares, Alexandre Leite R. de Oliveira, Cristina Pontes Vicente, Rui Seabra Ferreira, Benedito Barraviera, Maria Esméria C. do Amaral, Marcelo Augusto M. Esquisatto, Benedicto de Campos Vidal, Edson Rosa Pimentel, Andrea Aparecida de Aro

**Affiliations:** 1 Department of Structural and Functional Biology, Institute of Biology, University of Campinas–UNICAMP, Charles Darwin, s/n, CP 6109, 13083-970 Campinas, SP, Brazil; kafrauz@hotmail.com (K.F.); teo.luisfelipe@gmail.com (L.F.R.T.); gianedc@gmail.com (G.D.C.); daniloferrucci@yahoo.com.br (D.L.F.); aobombeiro@gmail.com (A.L.B.); alroliv@unicamp.br (A.L.R.d.O.); crpvicente@gmail.com (C.P.V.); camposvi@unicamp.br (B.d.C.V.); pimentel@unicamp.br (E.R.P.); 2 Department of Biochemistry and Tissue Biology, Institute of Biology, University of Campinas–UNICAMP, Charles Darwin, s/n, CP 6109, 13083-970 Campinas, SP, Brazil; fernandaveiga6@gmail.com (F.C.d.V.); lealvare@unicamp.br (L.E.A.); 3 Engineering, Modeling and Applied Social Sciences Center (CECS), Biomedical Engineering Graduate Program (PPGEBM), Universidade Federal do ABC (UFABC), Alameda da Universidade s/n, 09606-045 São Bernardo do Campo, SP, Brazil; pritsimoes@gmail.com; 4 Center for the Study of Venoms and Venomous Animals (CEVAP), São Paulo State University (UNESP – Universidade Estadual Paulista), Botucatu, SP, St. José Barbosa de Barros, 1780, Fazenda Experimental Lageado, 18610-307 Botucatu, SP, Brazil; rui.ead@gmail.com (R.S.F.J.); bbviera@jvat.org.br (B.B.); 5 Biomedical Sciences Graduate Program, Herminio Ometto University Center-UNIARARAS, Av. Dr. Maximiliano Baruto, 500, Jd. Universitário, 13607-339 Araras, SP, Brazil; esmeria@fho.edu.br (M.E.C.d.A.); marcelosquisatto@fho.edu.br (M.A.M.E.)

**Keywords:** repair, tenomodulin, collagen, birefringence, scaffold

## Abstract

Tissue engineering and cell-based therapy combine techniques that create biocompatible materials for cell survival, which can improve tendon repair. This study seeks to use a new fibrin sealant (FS) derived from the venom of *Crotalus durissus terrificus*, a biodegradable three-dimensional scaffolding produced from animal components only, associated with adipose-derived stem cells (ASC) for application in tendons injuries, considered a common and serious orthopedic problem. Lewis rats had tendons distributed in five groups: normal (N), transected (T), transected and FS (FS) or ASC (ASC) or with FS and ASC (FS + ASC). The in vivo imaging showed higher quantification of transplanted PKH26-labeled ASC in tendons of FS + ASC compared to ASC on the 14th day after transection. A small number of Iba1 labeled macrophages carrying PKH26 signal, probably due to phagocytosis of dead ASC, were observed in tendons of transected groups. ASC up-regulated the *Tenomodulin* gene expression in the transection region when compared to N, T and FS groups and the expression of *TIMP-2* and *Scleraxis* genes in relation to the N group. FS group presented a greater organization of collagen fibers, followed by FS + ASC and ASC in comparison to N. Tendons from ASC group presented higher hydroxyproline concentration in relation to N and the transected tendons of T, FS and FS + ASC had a higher amount of collagen I and tenomodulin in comparison to N group. Although no marked differences were observed in the other biomechanical parameters, T group had higher value of maximum load compared to the groups ASC and FS + ASC. In conclusion, the FS kept constant the number of transplanted ASC in the transected region until the 14th day after injury. Our data suggest this FS to be a good scaffold for treatment during tendon repair because it was the most effective one regarding tendon organization recovering, followed by the FS treatment associated with ASC and finally by the transplanted ASC on the 21st day. Further investigations in long-term time points of the tendon repair are needed to analyze if the higher tissue organization found with the FS scaffold will improve the biomechanics of the tendons.

## 1. Introduction

Tendons are load-bearing structures, which transmit muscle-contraction force to the skeleton so it can maintain posture or produce motion [1]. The abundant tendon extracellular matrix (ECM) is formed by a hierarchical collagen structure composed specially by type I collagen produced by tenocytes, which are associated with numerous non-fibrillar proteins, that are essential to tendons ability of supporting load with stability [2]. The specific mechanical properties of tendons are directly related to the high organization of collagen bundles [3], so as more organized, more resistant to tensile load. As known, any external mechanical load stimulates a mechanotransduction mechanism [4]. It is important for homeostasis and tendon structural integrity as well as for the modulation of synthesis and degradation of ECM components produced by tenocytes [5]. The load transmits different levels and combinations of tensile and compressive forces [6] to tendons and the precise physiologic loads of individual tendons depending on their function, age, sex, location and species [5]. However, abnormal loading can cause tendon injury due to an acute traumatic injury or to degenerative processes [7,8].

Tendon injuries are among the most common orthopedic problems with long-term disability as a frequent consequence due to its prolonged healing time. Further, the repaired tissue presents lower biomechanical resistance, predisposing patients to high rates of recurrence ensuing initial injury [9]. Following an acute rupture, the tendon undergoes a healing process, involving successive steps of inflammation, ECM formation and remodeling [10,11]. Yet, the scar tissue formed during tendon repair is different from the native tendon, presenting one-third of tensile strength observed in native tendons [12].

Several strategies have been studied aiming the tendon repair process to be more effective, attempting to form similar tissue to native tendon. Currently, tissue engineering and cell-based therapy combine techniques to create biocompatible materials for cell survival, which can improve the tissue repair. Considering this field of regenerative medicine based on the cell-based therapy, the investigation of the effects of adipose-derived stem cells (ASC) during wound healing has grown immensely in recent years, due to its high responsiveness to distinct environmental cues and isolation facility [13,14]. Thus, a Gonçalves et al. [15] study confirmed the potential of ASC for tendon regeneration, so proving the role of some growth factors such as EGF (epidermal growth factor), bFGF (basic fibroblast growth factor), PDGF (platelet derived growth factor) and TGF-b1 (beta-1 transforming growth factor) in the tenogenic differentiation of ASC.

The ASC are a population of multipotent cells that can be obtained from subcutaneous adipose tissue through percutaneous or limited open aspiration techniques [16,17]. Under the appropriated conditions, ASC hold the direct differentiation potential towards specific cells lineages as fibroblasts [18,19], osteoblasts, chondroblasts, adipocytes and myoblasts [20,21,22]. ASC produce important molecules, which play an important role in wound repair as growth factors [23,24], cytokines, matrix metalloproteinases (MMP) [25] and collagen [26,27]. Still, it is extremely small the percentage of ASC that survives after transplantation into a site of tissue injury [28], demonstrating the importance of new scaffolds that can create a good environment for cell functionality. The tenogenic differentiation potential as well as the paracrine secretion of ASC at the injury site during tendon repair have not been extensively described in the literature.

Ideal biodegradable scaffolds for cells should provide them mechanical support, cells adhesion, proliferation and cellular differentiation [29]. According to James et al. [30], scaffolds for tendons should have high porosity, a large surface area and they should also mimic the native tendon ECM architecture, allowing nutrients diffusion and factors secreted by the cells, important for the stimulus of cell proliferation and synthesis of the ECM’s components during tissue repair. In the present study, a new fibrin sealant (FS) derived from snake venom from *Crotalus durissus terrificus* was used with a biological three-dimensional scaffolding capacity of maintaining cell survival without interfering in its differentiation and with cell viability rates above 80% [29]. Gasparotto et al. [29] showed an excellent interaction of this FS with the ASC, due to its ability to induce the spontaneous adipogenic, chondrogenic and osteogenic lineages differentiation. This new FS is composed of a fibrinogen-rich cryoprecipitate extracted from the *Bubalus bubalis* buffalo’s blood in association with a serine protease (a thrombin-like enzyme) extracted from *Crotalus durissus terrificus* venom [30,31,32,33]). According to Ferreira et al. [34], a thrombin-like enzyme, in the presence of calcium, acts upon the fibrinogen molecule transforming it into fibrin monomers forming a stable clot with adhesive, hemostatic and sealant effects [32,33,35].

Fibrin has been used for many years specially because it presents important characteristics like adhesive tissue or sealant to control bleeding, being used for a variety of surgical and repairing processes [29,36,37]. FS has positive effects for bone [38] and cardiac [39] tissue engineering, for peripheral nerve [40] or skin repair [41] among other applications. Still, concerns about the risk transmission of some viral diseases of commercial FS have increased researchers’ interest to develop new sealants [34]. Then, the new FS used in the present study has advantages when compared to the commercially available FS products, since it is produced from animal components only, without risk of infectious diseases and lower costs of production [29].

Through the hypothesis of FS being a good scaffold for ASC, as much for tendon graft considering the FS malleability, which is important during limb movement in our model of tendon transection, the goals of this study are: (1) to evaluate the presence of ASC in the FS at the transected region of the tendons until the 21st day after injury; (2) to analyze the cells paracrine secretion through the expression of genes related to tendon remodeling; (3) to measure the organization of the collagen fibers and to quantify the total collagen content; and (4) to test the biomechanical properties of tendons.

## 2. Materials and Methods

### 2.1. Isolation of ASC and Ccell Culture

The procedure was done according to Yang et al. [42] with some modifications. Adipose tissue was obtained from the inguinal region of 10 male Lewis rats between 90–120 days. All surgical and experimental protocols were approved (01/12/2015) by the Institutional Committee for Ethics in Animal Research of the State University of Campinas-UNICAMP-Brazil (Protocol nº 3695-1). Adipose tissue was cut and washed in Dulbecco’s modified phosphate buffered saline solution (DMPBS Flush without calcium and magnesium) containing 2% streptomycin/penicillin. Then, 0.2% collagenase (Sigma-Aldrich^®^ Inc., Saint Louis, MO, USA) was added to ECM degradation and the solution was maintained at 37 °C under gentle stirring for 1 h to separate the stromal cells from primary adipocytes. Dissociated tissue was filtered using cell strainers (40 μm) and the inactivation of collagenase was then done by the addition of equal volume of Dulbecco’s modified Eagle’s medium (DMEM) supplemented with 15% fetal bovine serum (FBS), followed by centrifugation at 1800 rpm for 10 min. The suspending portion containing lipid droplets was discarded and the pellet was resuspended in DMEM with 15% FBS and transferred to 25 cm^2^ bottle. After confluence, cells were transferred to 75 cm^2^ bottle (1st passage) and the cultures were maintained at 37 °C with 5% CO_2_ until the 5th passage (5P). For detachment of the adherent cells, it was used 0.25% trypsin-0.02% EDTA and re-plated at a dilution of 1:3.

### 2.2. Flow Cytometry

ASC at 5P (*n* = 4) were trypsinized and centrifuged at 1800 rpm for 10 min and counted using the Neubauer chamber. 1 × 10^6^ ASCs were resuspended in 200 μL of DMPBS with 2% BSA (bovine serum albumin). For the immunophenotypic panel [29,43], the following antibodies were used: CD90-APC (eBioscience^®^ Inc., San Diego, CA, USA), CD105-PE (BD-Pharmingen^TM^, San Diego, CA, USA) and CD34-FITC double conjugated (eBioscience^®^ Inc., San Diego, CA, USA), were diluted 1:200 and incubated with cells during 1 h at room temperature. Subsequently, ASCs were washed twice with 500 μL of DMPBS and centrifuged at 2000 rpm for 7 min. The ASCs were resuspended in DMPBS with 2% BSA, following for flow cytometry analysis.

### 2.3. In Vitro Differentiation Potential of ASC

ASC (5P) were cultured (2 × 10^4^ cells) using different media for each type differentiation, according to Yang et al. [42]. Osteogenic differentiation: DMEM supplemented with 10% FBS, 0.1 μM dexamethasone, 200 μM ascorbic acid, 10 mM β-glycerol phosphate. Adipogenic differentiation: DMEM supplemented with 10% FBS, 1 μmol/L dexamethasone, 50 μmol/L indomethacin, 0.5 mM 3-isobutyl-1-methyl-xanthine and 10 μM insulin. Chondrogenic differentiation: DMEM supplemented with 10% FBS, acid free 15 mM HEPES, 6.25 μg/mL insulin, 10 ng/mL TGF-β1 and 50 nM ascorbic acid-2-phophate. Cultures were maintained at 37 °C with 5% CO_2_ and the complete mediums were replaced twice a week, for four weeks. At the end of each culture differentiation, the cells were fixed with 4% paraformaldehyde for 20 min and stained with 2% Alizarin Red S (pH 4.1) during 5 min for calcium detection, with 0.025% Toluidine blue in McIlvaine buffer (0.03 M citric acid, 0.04 M sodium phosphate dibasic—pH 4.0) during 10 min for proteoglycans detection and with 1% Sudan IV during 5 min to show lipids droplets. Samples were imaged on the Axiovert S100 (ZEISS) (Carl Zeiss AG, Oberkochen, Germany) inverted microscope.

### 2.4. Fibrin Sealant (FS) Scaffold

The FS derived from serpent venom (*Crotalus durissus terrificus*) was kindly provided by the Center for the Study of Venoms and Venomous Animals at UNESP (CEVAP at UNESP, São Paulo State University—Brazil) and its components and instructions for use are described in its patents (registration numbers BR1020140114327 and BR1020140114360). This FS, which was developed by the researchers of CEVAP, was produced according to the proposed standardization [29,33,35,44,45]. This new sealant was produced from the thrombin-like enzyme extracted from snake venom and animal fibrinogen. The product was provided in three microtubes that were stored at −20 °C. At the time of use, the components were previously thawed, reconstituted, mixed and applied (9 μL in each transected tendon) to generate a stable clot with a dense fibrin network.

### 2.5. Confocal Microscope Analysis

Approximately 3.7 × 10^5^ from 1 bottle of 75 cm² ASC on 5P (*n* = 3) were trypsinized and centrifuged at 1800 rpm for 10 min. The supernatant was discarded and the pellet containing ASC was fixed with 4% paraformaldehyde. ASC were labeled with phalloidin-FITC at 1.25 μg/mL for 5 min at room temperature to stain the actin cytoskeleton and incubated with DAPI (0.1 mg/mL in methanol) for 5 min at 37 °C to stain the nucleus. Then, the ASC were resuspended in 15 μL of DMPBS + 9 μL of FS and, after clot formation, were analyzed in the National Institute of Science and Technology on Photonics Applied to Cell Biology (INFABIC) at the State University of Campinas (UNICAMP), using a Zeiss LSM 780-NLO confocal on an Axio Observer Z.1 microscope (Carl Zeiss AG, Oberkochen, Germany) using a 10× objective. Images were collected using laser lines 405 nm and 488 nm for excitation DAPI and phalloidin-FITC fluorophores, respectively, with pinholes set to 1 airy unit for each channel, 1024 × 1024 image format.

### 2.6. Experimental Groups

A total of 110 male Lewis rats (120-day-old) kept at a constant temperature (23 ± 2 °C) and humidity (55%) under a 12/12 h light/dark cycle, with free access to food and water, were divided into 5 experimental groups: Normal (N): rats with tendons without transection; Transected (T): rats with partially transected tendons and treated with topical application of DMPBS in the transected region; Fibrin sealant (FS): rats with transected tendons and treated with FS application in the transected region; Mesenchymal stem cells derived from adipose tissue (ASC): rats with transected tendons with subsequent transplant of ASC (3.7 × 10^5^ cells) in the transected region; And FS with ASC (FS + ASC): rats with transected tendons and treated with application of FS associated with ASC in the transected region. Animals were euthanized on the 21st day after transection by an overdose of anesthetic (ketamine and xylazine). Animals of the N group were euthanized at 141 days and the tendons without transection were collected for analysis.

### 2.7. Protocol for Partial Transection of the Achilles Tendon and Application of FS and ASC

The animals were anesthetized with intraperitoneal injection of Ketamine (90 mg/Kg) and Xylazine (12 mg/Kg) and the right lower paws submitted to antisepsis and trichotomy. To expose the calcaneus tendon, a longitudinal incision was made in the animal’s skin, followed by transverse partial transection performed in the proximal tendon region (predominantly subjected to tension forces) located at a distance of 4 mm from its insertion in the calcaneus bone [11,46,47,48,49,50,51]. Approximately 3.7 × 10^5^ ASCs in the 5P were resuspended in 15 μL of DMPBS and transplanted in the transected region of tendons in the ASC group, using a pipette. In the FS + ASC group, the ASCs were resuspended in 15 μL of DMPBS + 9 μL of FS and, after clot formation; it was placed on top of the transected region. In each T-group tendon only 15 μL of DMPBS was applied and the SF group tendons received application of 15 μL of DMPBS + 9 μL FS. All applications were made in the region of the tendon where partial transection was performed. Then, the skin was sutured with nylon thread (Shalon 5-0) and needle (1.5 cm). All surgical and experimental protocols were approved (01/12/2105) by the Institutional Committee for Ethics in Animal Research of the UNICAMP—Brazil (Protocol nº 3695-1).

### 2.8. In Vivo Imaging

The ASC on 5P were trypsinized and centrifuged at 1800 rpm for 10 min. The supernatant was discarded and the pellet containing about 3.7 × 10^5^ cells were labeled with PKH26 (Sigma) as previously described [52]. After labeling with PKH26 the pellet was resuspended in 15 μL of DMPBS and applied to the transected region of the calcaneus tendon of the ASC group. In the FS + ASC group the same procedure was repeated with the addition of 9 μL of FS. In the control rat, sham ASC were applied on the intact tendon and the negative control rat had only its tendon transected without ASC or FS application. In vivo imaging was performed on the 1st, 2nd, 3rd, 7th, 14th and 21st days after injury using the In vivo FX PRO device (BRUKER^®^, Billerica, Massachusetts, USA) for identification and quantification of the intensity and area of fluorescence of the ASC labeled with PKH26. To this end, the wavelengths of 550 nm and 600 nm were applied for excitation and emission of the fluorophore, respectively, for 1 min. In addition, the animals were submitted to X-ray, aiming at the anatomical location of the marking. During the procedure, the animals of experimental groups (*n* = 3) were kept under anesthesia (3% isoflurane in medicinal air). In order to exclude possible nonspecific markings, negative control was also subjected to imaging. Additionally, the fur was completely removed from the area of interest and the skin was cleaned with 70% ethanol to remove any residues that could interfere with the fluorescence. The following parameters were analyzed: fluorescence area (mm²) and fluorescence intensity (photons per second per square millimeter, P/s/mm^2^).

### 2.9. Real-Time PCR Array

The tendons were collected carefully (*n* = 4), placed in stabilizing solution (RNA-later, QIAGEN^®^, Hilden, Germany) and maintained at −20 °C. For total RNA extraction, the transected region (TR) of tendons were isolated and sprayed using liquid nitrogen and then homogenized in a tube containing 5 stainless steel balls (2.3 mm diameter, Biospec Products, Inc., Bartlesville, USA) by being shaken in TissueLyser LT instrument (QIAGEN^®^), with 2 repetitions (60 s) intercalated with ice cooling (2 min) between each shaking step [53]. Total RNA was in isolation from each sample using the RNeasy^®^ Fibrous Tissue Mini Kit (QIAGEN^®^), following the manufacturer’s instructions. A spectrophotometer (NanoDrop^®^ ND-1000, Thermo Scientific^®^, Waltham, Massachusetts, USA) was used to quantify RNA in each sample by determining the absorbance ratio at 260 and 280 nm. 0.5 μg from the total extracted RNA of each sample was used for the synthesis of cDNA, using the RT^2^ First Strand Kit (QIAGEN^®^) and thermocycler Mastercycler Pro (Eppendorf^®^, Hamburg, Germany), also following the manufacturer’s instructions. The cDNA was frozen at −20 °C until tested. The RT-PCR array reaction was performed using the RT^2^ Profiler PCR Arrays (A format) kit in combination with the RT^2^ SYBR Green Mastermixes (QIAGEN^®^) on the thermocycler apparatus 7300 (ABI Applied Biosystems^®^, Foster City, CA, USA), following the manufacturer’s instructions. For each animal sample, three types of reaction controls were used: 1. Positive PCR control; 2. Reverse transcriptase control; 3. Control for contamination of rat genomic DNA. The *Glyceraldehyde-3-phosphate dehydrogenase* (*Gapdh*, NM_017008) was used as endogenous control for each sample. The following genes were analyzed (QIAGEN^®^): *Scleraxis* (*Scx*, NM_001130508); *Tenomodulin* (*Tnmd*, NM_022290); *Tumor necrosis factor* (*TNF superfamily, member 2*) (*Tnf*, NM_012675); *Interleukin 1 beta* (*II1b*, NM_031512); *Transforming growth factor, beta 1* (*Tgfb1*, NM_021578); *Matrix metallopeptidase 2* (*Mmp2*, NM_031054); *Matrix metallopeptidase 9* (*Mmp9*, NM_031055); *TIMP metallopeptidase inhibitor 2* (*Timp2*, NM_021989); *Decorin* (*Dcn*, NM_024129); *Lysyl oxidase* (*Lox*, NM_017061) e *Growth differentiation factor 5* (*Gdf5*, XM_001066344). Reactions were made in a single cDNA pipetting for each gene including endogenous control. ∆CT values were obtained by the difference between the CT values of the target genes and the *Gapdh* gene. These values were normalized by subtracting the ∆CT value of the calibrator sample (N group) to obtain ∆∆CT values. For each target gene, the 2^−∆∆CT^ method was used to calculate the relative expression level (fold change) and the results were represented as the relative gene expression in comparison to the calibrator sample that is equal to 1.

### 2.10. Dosage of Hydroxyproline

Hydroxyproline was used as an indicator of total collagen amount in tendons of different groups (*n* = 8) used previously in the biomechanical assay. The entire tendons were cut and immersed in acetone for 48h, also followed by a solution containing chloroform: ethanol (2:1) for 48h. After dehydration, the samples were hydrolyzed in 6N HCl (1 mL/10 mg of tissue) for 4h at 130 °C according to Stegemann and Stalder [54], with some modifications. The hydrolysate was neutralized with 6N NaOH, followed by spectrophotometric quantification, according to Jorge et al. [55]. The absorbance of the samples was measured at 550 nm using a microplate reader (Expert Plus, Asys^®^, Holliston, MA, USA).

### 2.11. Western Blotting

For protein extraction, 12 entire tendons longitudinal cryosections obtained from 4 different animals of each group were carried out using 50 μL of T-PER^TM^ Tissue Protein Extraction Reagent. The extraction mixture was gently stirred for 30 min at 4 °C, followed by centrifugation at 10,000 rpm for 10 min. The supernatant (total extract) was used for determination of the protein concentration by the biuret method. Aliquots of the supernatant were treated with Laemmli buffer containing 100 mM DTT (Sigma). Samples containing 30 µg of protein were boiled for 5 min and loaded onto 6.5% to 10% SDS-PAGE gels. The gels were run in a Mini-Protean apparatus (Bio-Rad, Hercules, CA, USA) and transferred to PVDF membranes (Bio-Rad). The membranes were washed in basal solution (1 M Trizma base, 5 M NaCl, 0.005% Tween 20 and deionized water) and incubated in blocking solution (basal solution plus 5% Molico skim milk) for 2h. Then, the membranes were incubated with a polyclonal antibody against collagen type I (1:1000; C2456-Sigma), Tenomodulin (1:1000; SAB2108237-Sigma) and Beta-actin used as internal control (1:500; sc-47778-Santa Cruz Biotechnology, California, CA, USA), overnight at 4 °C. Specific protein bands were visualized in the PVDF (Bio-Rad^®^) membranes incubated with appropriate secondary antibodies at 1:10,000 (Santa Cruz Biotechnology, California, CA, USA) for 2h, followed by exposure to the SuperSignal West Pico Chemiluminescent Substrate kit. Membranes were developed with the Syngene G: BOX documentation system. The band intensities were quantified by optical densitometry, using the free Image J software (National Institutes of Health, Bethesda, MD, USA).

### 2.12. Preparation of Sections in Freezing

Tendons were placed in Tissue-Tek^®^, frozen and cut in cryostat (serial longitudinal cuts of 7 μm thickness). The sections were fixed using a 4% formaldehyde solution in Millonig buffer (0.13 M sodium phosphate and 0.1 M sodium hydroxide, 7.4 pH) for 20 min and followed for birefringence and contrast analysis by differential interference and for histology and histomorphometry.

### 2.13. Birefringence and Contrast by Differential Interference (DIC)

For this procedure the Olympus BX-51 (Olympus America, Center Vallery, PA, USA) equipped with Q-color 5 camera (Olympus America, Center Vallery, PA, USA) was used. For visual evaluation and birefringence measurements, 3 tendons longitudinal cryosections obtained from 5 different animals of each group were carried out and Image a Pro-plus v.6.3 software for Windows™ (Media Cybernetics, Silver Spring, MD, USA) was used. Around 300 measurements were done in tendon sections of each group. With the microscope and the software it is possible to carry out analysis of DIC and anisotropic properties, both individually and in combination [56,57]. To obtain the birefringence of the fibers and collagen bundles without Walston’s prism activity, these were removed from the polarized light path by turning the condenser tower to the position of a field of observation free, giving passage only to polarized light. The Nomarski prism was also removed below the analyzer. Under these conditions we simply have a polarizing microscope. In place of the Nomarski prism a Senarmont compensator of 1/4 wavelength was inserted and used for collagen bundles analysis before and after compensation. After studying the fibers morphology and collagen bundles by their birefringence of sections immersed in distilled water, birefringence was measured by captured images analysis. To do this, a standardization of the lighting source and camera sensitivity was used so that the same working conditions were always preserved. In optical terms, 40 × objective and monochromatic light with λ = 546 nm were always used. Longitudinal tendon sections of all groups were examined and measured. As standard to adjust the working conditions, sections of the ASC group were adopted as treatment from which a hypothetically ideal response was expected. The birefringence measurements were in terms of image brightness and expressed in pixels, which allowed a high sampling of measured areas, as well as the detection of the greater variability of birefringence as previously verified [58,59].

### 2.14. Immunofluorescence

Tendon longitudinal cryosections (*n* = 5) were fixed in acetone (4 °C, 20 min) and washed with PBS (2 × 5 min). For ASC labeling, sections were blocked with PBS/1% BSA (1h, room temperature) and then incubated with anti-rat CD90/CD90.1 (0.5mg/mL, BD-Pharmingen) diluted in PBS/1% BSA (1:200, 2h, room temperature). After washed in PBS (2 × 5 min), sections were incubated with anti-rat FITC (0.5mg/mL, BD-Pharmingen) and PE rat anti-mouse CD105 (0.2mg/mL, BD-Pharmingen), diluted in PBS (both 1:200, 40 min, room temperature). Immediately after washed in PBS (2 × 5 min), the sections were incubated with DAPI (0.1 mg/mL in methanol) for 5 min at 37 °C. The sections were analyzed in a fluorescence microscope (Olympus BX60) and the images were captured by the Q-Capture Pro™ software (QImaging, Surrey, BC, Canada). For macrophages, sections were fixed as above, blocked with PBS/3% BSA (1h, room temperature) and then incubated with anti-Iba1 rabbit IgG (Wako, cat. code: 019-19741) diluted in PBS/1% BSA (1:700, overnight, 4 °C). After washings (3 × 5 min in PBS), sections were incubated with CY2 donkey anti-rabbit IgG (Jackson Immunores., cat. code: 711-225-152) diluted in PBS/1% BSA (1:500, 45min, room temperature). Sections were washed in PBS (3 × 10 min) and then coverslips were mounted using glycerol solution (glycerol and water, 3:1) containing DAPI (1:1000). Slides were photographed in a fluorescence microscope (Leica DM5500B with digital camera Leica DFC345 FX, using Leica Application Suite X software, Leica Microsystems GmbH, Wetzlar, Germany). For orthogonal sectioning (where indicated) the z stack varied from 20 to 30 layers and when necessary the 3D deconvolution was employed to those projections (total interactions: 10; refractive index: 1.52).

### 2.15. Histology and Histomorphometry

After fixing the tissue cryosections as described previously, tendons were stained with 0.025% toluidine blue (TB) in McIlvaine buffer (0.03 M citric acid, 0.04 M sodium phosphate dibasic-pH 4.0) [48] for proteoglycans observation and with hematoxylin and eosin (HE) [49] for a panoramic view of the tendon’s morphology. The sections on slides were air dried and immersed in xylene, before embedding in entellan (Merck, Rio de Janeiro, Brazil). Tissue sections were analyzed for tendon morphology observation under an Olympus BX53 microscope (Center Vallery, PA, USA).

For the fibroblast count (number of fibroblasts in 10^4^ µm^2^) of the transected region, longitudinal sections of tendons stained with Harris hematoxylin were used. Three tendons sections from each different animal group (*n* = 3) were used, in which five samples were taken for fibroblasts count. All images were captured and scanned using the Leica DM2000 Photomicroscope. Measurements were performed on scanned images supported by the SigmaScan Pro 5.0™ program (Systat Software Inc., Chicago, USA).

### 2.16. Biomechanical Parameters

Tendons from experimental groups (*n* = 8) were collected and stored at −20°C until tested. Before the biomechanical test, the tendons were thawed and measured with a pachymeter, considering their length, width and thickness. For the biomechanical assay, the tendons were maintained in PBS to prevent their fibers from drying out. Then, the tendons were fixed to metal claws by the myotendinous junction and by the osteotendinous junction, for a correct alignment of the equipment (Texturometer, MTS model TESTSTAR II). In each biomechanical assay tendons were subjected to a gradual increase of load at a displacement velocity of 1 mm/s by using a load 0.05 N, until the tendon ruptured. Biomechanical parameters were analyzed according to Biancalana et al. [60], such as maximum force (N) and maximum displacement (mm), which were used to calculate the maximum stress (Mpa) and maximum strain (L) of tendons from the experimental groups. The cross-sectional area of the calcaneus tendon was calculated by assuming an elliptical approximation (A = πWd/4), using measurements of width (W) and thickness (d) and values from the same [61]. The maximum stress value (MPa) was estimated by the ratio between the maximum load (N) and the cross-sectional area (mm^2^). The maximum deformation (L) was calculated through (L = L_f_ − L_i_/L_i_,), where (L_f_) is the value of the final length before rupture and (L_i_) is the initial tendon length value.

### 2.17. Statistical Analysis

All results were presented in mean and standard deviation for the values with normal distribution (or interquartile range and median for the values that did not adhere to the Gaussian distribution). For the data with normal distribution, it was used the analysis of variance (ANOVA), followed by the Tukey post-hoc test for intra-group analysis (in the case of statistical significance) or the Student’s T Test preceded by Levene’s Test (for biomechanical parameters, dosage of hydroxyproline, in vivo imaging, histomorphometry, real-time PCR array and Western blotting). For data that did not adhere to the Gaussian distribution, it was used non-parametric test by the Kruskal-Wallis test followed by the post-hoc test of Dunn for intra-group analysis (in the case of statistical significance) or U Test of Mann-Whitney (for birefringence measurements). Statistical analysis was performed in the software Statistical Package for Social Sciences (SPSS Inc., Chicago, Illinois, USA) version 22.0 and for all the aforementioned tests the significance level α = 0.05 and power of the test of 95% were considered.

## 3. Results

### 3.1. In Vitro Adipogenic, Chondrogenic and Osteogenic Differentiation of ASC and Positive ASC Markers

**Figure 1:**
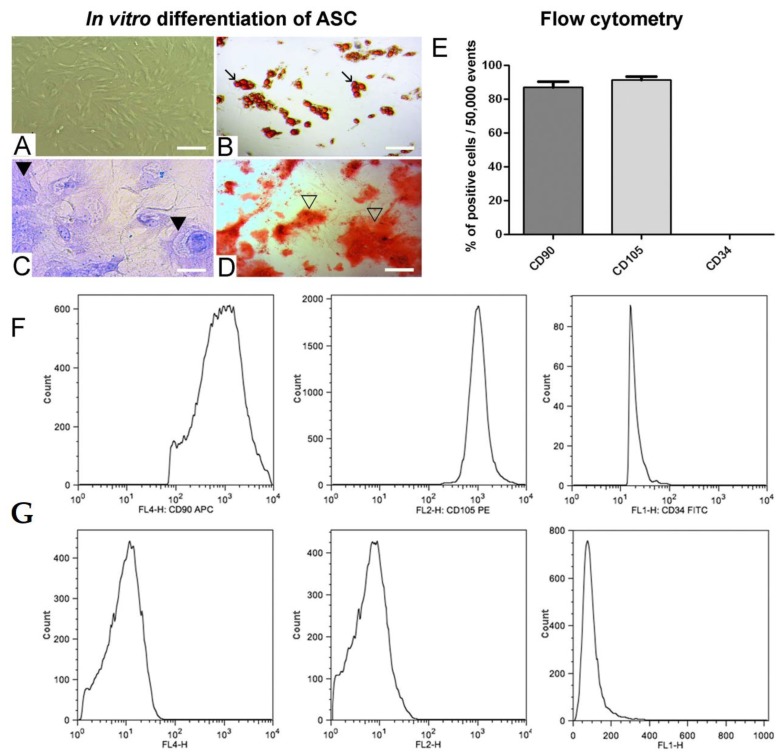
In vitro differentiation potential of ASC (*n* = 4) in 5P (**A**): adipogenic (**B**) and lipid stained with Sudan IV (→); chondrogenic (**C**) and proteoglycans stained with toluidine blue (▶); osteogenic (**D**) and calcium stained with alizarin red (▷). Different cells were stained after 4 weeks of culture. (**E**) Flow cytometry for ASC in 5P (*n* = 4) with positive labeling for CD90 and CD105 and negative labeling for CD34. (**F**) Histograms demonstrate the x-axis fluorescence scale considered positive when the cell peak is above 101 (CD34) or 102 (CD90 and CD105). (**G**) Control for -APC, -PE and -FITC (with very low fluorescence), corresponding to non-marked cells. Bars = A, B, D: 120 μm; C: 40 μm.

In vitro ASC (5P) staining with Toluidine Blue, Alizarin Red and Sudan IV showed differentiation in chondrocytes, osteoblast and adipocytes, respectively (Figure 1A–D). In flow cytometry, CD90 positive cells (approximately 87%) and CD105 positive cells (approximately 91%) were observed. CD34 cells were not detected (Figure 1E,F).

### 3.2. ASC Disposition in the FS and Application on the Transected Tendon

**Figure 2:**
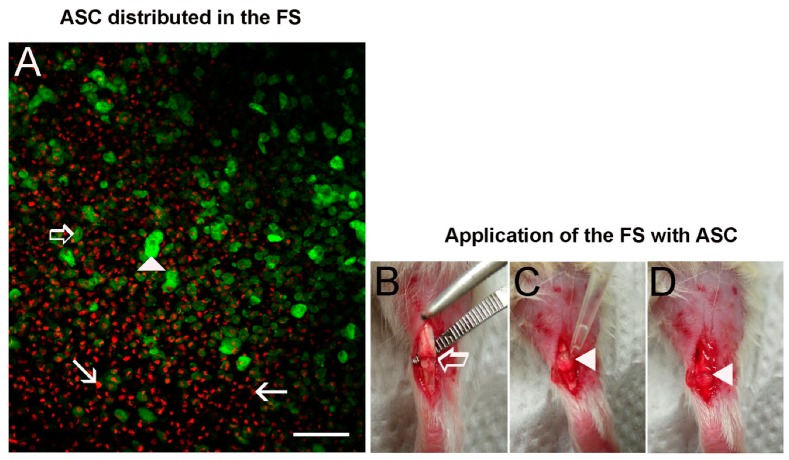
(**A**) Confocal microscope image of ASC labeled with phalloidin-FITC (actin cytoskeleton in green) and DAPI (nucleus in red) distributed in the dense fibrin network formed by the FS (*n* = 3). Observe the disposition of 3.7 × 10^5^ cells in the FS before application in the tendon transected region. (▶) superficial cells, (⇨) intermediately positioned cells and cells at the bottom (→). (**B**) Model of tendon injury showing the partial transection (⇨) in the proximal region of the Achilles tendon. (**C**) Application of the FS with ASC using a pipette: note the formation of a clot (▶). (**D**) Representation of the FS with ASC (▶) covering the transected region before the skin suture. Bar = 200 μm.

A disposition of approximately 3.7 × 10^5^ ASC in the dense fibrin network formed by the FS was analyzed, showing the homogenous distribution of the cells in the fibrin clot (Figure 2A) before transplantation in the transection tendon region (Figure 2B–D).

### 3.3. In Vivo Imaging for ASC Detection on Tendon

**Figure 3:**
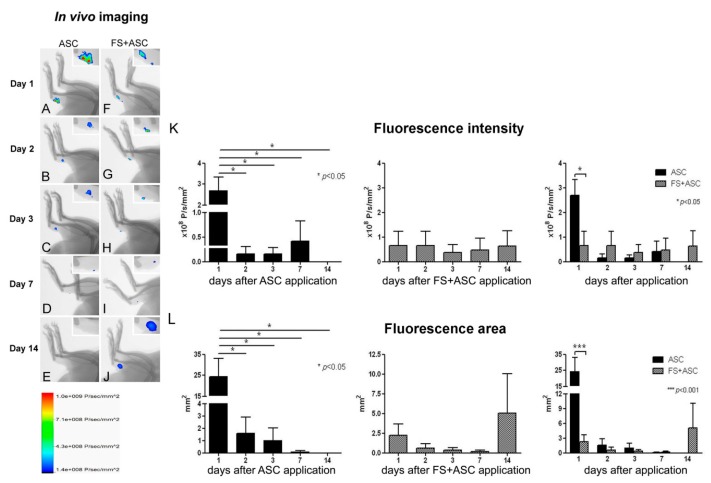
(**A**) In vivo imaging for detection and quantification of PKH26-labeled ASC in the tendon transected region: ASC (**A**–**E**) and FS + ASC (**F**–**J**) groups were analyzed on the 1st, 2nd, 3rd, 7th,14th and 21st days after injury (*n* = 3). Observe the fluorescence intensity and area in the detail of each image of tendons (**A**–**J**). Scale: fluorescence intensity (×10^8^ P/s/mm²). Fluorescence intensity (**K**) and fluorescence area (**L**) occupied by ASC after quantification of images from in vivo imaging. Significant difference represented by (*) and (***) between the ASC and FS + ASC groups.

Cell migration was evaluated by in vivo imaging of animals that were injected with ASC labeled with PKH26. PKH26-labeled ASC (group ASC) or with fibrin sealant (group FS + ASC) were observed in lower limb up to 7 days (Figure 3A–D,F–I) or 14 days (Figure 3E,J), respectively, being not observed in any of the groups at the experiment end point (21 days, not shown). A small spot was seen in the sham group but never in the negative control, only on the 1st day (not shown), then its area was discounted from the other groups at the same day. According to our data, labeling intensity peaked at day 1 in the ASC group, decreasing on the 2nd day (1d vs. 2d, *p* < 0.05) with no further variation, while in the FS + ASC group it kept constant through time (Figure 3K). Of importance, comparison of both groups revealed higher fluorescence intensity in ASC treated rats on the 1st day (*p* < 0.05; Figure 3K). Regarding the labeling area, it also peaked on the 1st day in the ASC group, decreasing 93.5% on the 2nd day (1d vs. 2d, *p* < 0.05) after when it oscillated up to 7th day, however, with no significant differences (Figure 3L). No labeling was found on the 14th day (Figure 3E). Variations in the labeling area were also observed in the FS + ASC group from the 1st to the 14th day, however, with no statistical differences (Figure 3L). Comparison between both groups revealed that the labeling area is more than 10 folds higher in the absence of fibrin sealant on the 1st day (*p* < 0.001; Figure 3L), reinforcing its importance as a scaffold that avoids cell spreading in the tissue. No PKH26-labeled ASC was observed on the 21st day after tendon transection in both ASC and FS + ASC groups (not shown).

### 3.4. Cell Migration Assay and Macrophages Identification

**Figure 4:**
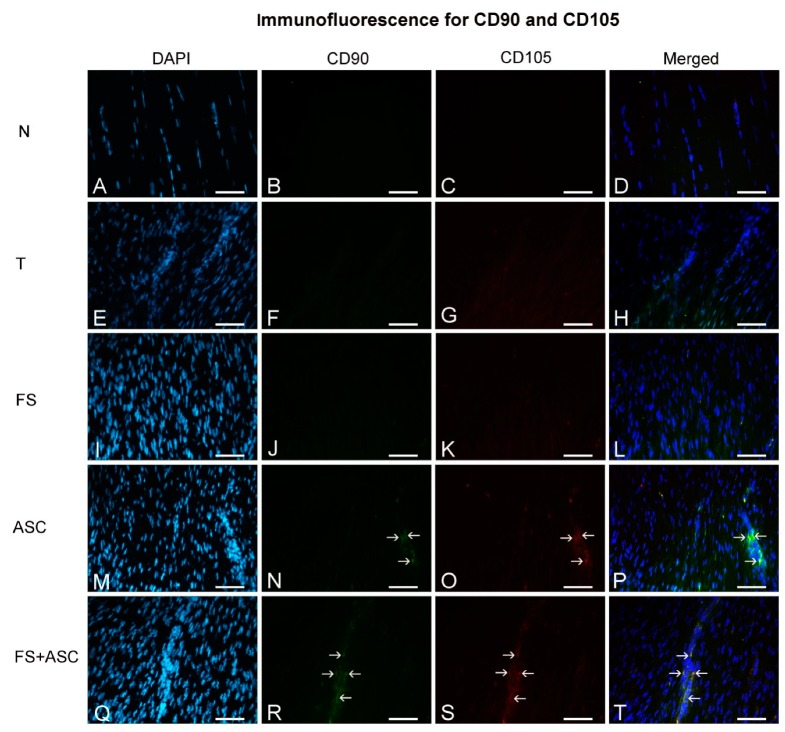
Immunofluorescence for CD90 and CD105 observed in the central portion of the TR of tendons on the 21st day (*n* = 5). Groups N (**A**–**D**), T (**E**–**H**), FS (**I**–**L**), ASC (**M**–**P**) and FS + ASC (**Q**–**T**). Note CD90 and CD105 (→) positive marking in the transected region of ASC and FS + ASC groups. Bar = 50 μm.

CD90+CD105+ ASC were visualized in the transected region of tendons of both ASC and FS + ASC groups (Figure 4), proving the ASC migration.

**Figure 5:**
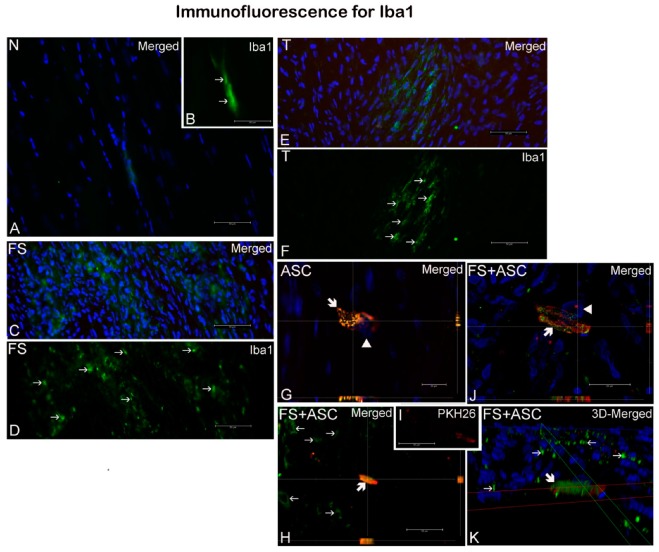
Iba1 labeled macrophages (green, →) were observed in all tendons (*n* = 3) regardless the treatment as follows: N group (**A**, **B**), FS (**C**, **D**), T (**E**, **F**), ASC (**G**) and FS + ASC (**H**–**K**). Eventually, in groups that received PKH26-labeled ASC (ASC and FS + ASC) fluorescence signal (red) could be seen inside of macrophages (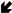). (**B**) Detail of A, showing Iba1 labeled macrophages. (**I**) Detail of H, evidencing PHK26 labeled ASC. (**G**, **H**, **J**) orthogonal sectioning; K, 3D deconvolution. Nuclei were stained with DAPI (blue, ▶). Scale bars: **A**–**F**, **H**, **I**, 50μm; **G**, **J**, 20μm.

A small amount of macrophages was observed in the normal (Figure 5A,B) as well as in the transected tendons regardless the treatment (Figure 5C–K). Of importance, in tendons that received cells it was possible to observe very few macrophages carrying PKH26 signal (Figure 5G,H,J,K), probably due to phagocytosis of dead ASC.

### 3.5. Genes Expression Analysis

**Figure 6:**
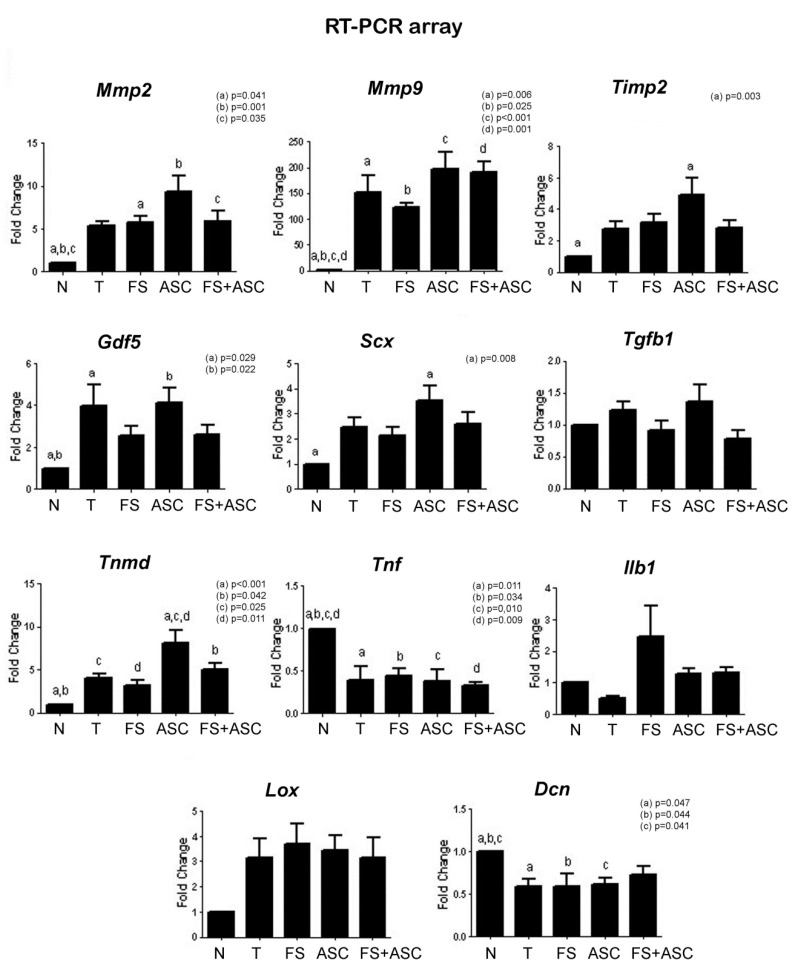
Real-time PCR array data of genes on the 21st day after the tendon transection (*n* = 4). Note the higher *Tnmd* expression in the ASC group in relation to the T and FS. Difference between N group and T, FS, ASC and FS + ASC groups can be observed for genes expression of *Mmp2*, *Mmp9*, *Timp2*, *Gdf5*, *Scx*, *Tnmd*, *Tnf* and *Dcn*. Same letters (a, b, c, d) = significant difference between the groups.

A higher expression level of the *Tnmd* gene could be observed in the ASC group in relation to T and FS groups. Trends to a greater expression of *Mmp2, Timp2, Scx* and Ilb1 were observed in the ASC group compared to the other transected groups. In comparison to N group, the transected groups presented higher expression of *Mmp2*, *Mmp9*, *Timp2*, *Gdf5*, *Scx* and *Tnmd* (Figure 6).

### 3.6. Total Collagen Quantification

Higher concentration of hydroxyproline was observed in the ASC group compared to the N group (Table 1).

### 3.7. Collagen I and Tnmd quantification

**Figure 7:**
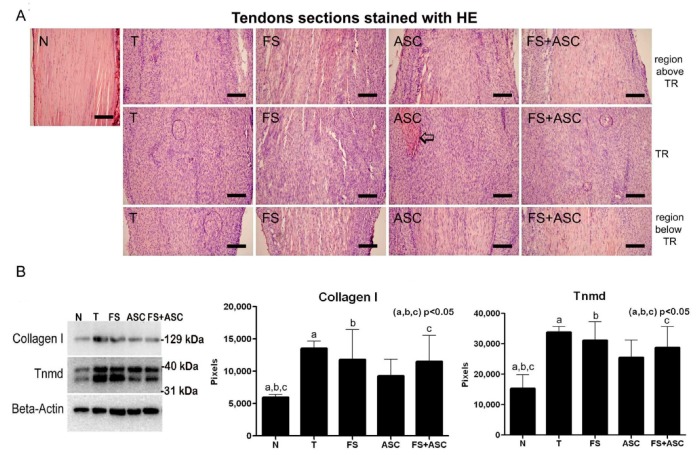
(**A**) Panoramic view of tendons sections stained with HE (*n* = 3). Comparing all transected tendons with normal tendon (proximal region), higher cellularity and light staining of ECM (**A**) can be seen in the TR and in the regions above and below TR. Note ECM more intensely stained in the TR of ASC (⇨). Both regions above TR (located closer to the insertion of tendon in the gastrocnemius muscle) and the region below TR (located closer to the insertion of tendon in the calcaneus bone) are located in the proximal region of the tendon. Bar = 200 μm. (**B**) Western blotting showing collagen type I and Tnmd in the entire tendon (*n* = 4). Beta-actin was utilized as an endogenous control (43 kDa). For the significant differences between the groups, see the band densitometry analysis in the graphics. The same letters (a, b, c) between the groups correspond to a significant difference between them.

The presence of collagen type I and Tnmd was observed in all groups. Densitometry analysis of the bands (pixels) showed a difference only between the normal tendon and the transected tendons, with exception of the ASC group (Figure 7B).

### 3.8. Panoramic View of Tendons Organization and Histomorphometry

The sections stained with HE showed few fibroblasts arranged between dense and organized matrix collagen bundles and characteristics of the proximal region of a normal tendon (Figure 7A). Higher cellularity and light staining of ECM were observed in the TR and in the regions above and below TR of transected tendons in relation to the normal tendon, indicating a strong remodeling process after lesion. The weak staining of collagen fibers by eosin shows that the reorganization of the matrix is not complete on the 21st day. Histomorphometry analysis showed no differences in the number of fibroblasts between the transected tendons. However, the N group presented a smaller number of cells compared to the T, FS, ASC and FS + ASC groups (Table 2).

### 3.9. Collagen Fibers Organization Measurements

**Figure 8:**
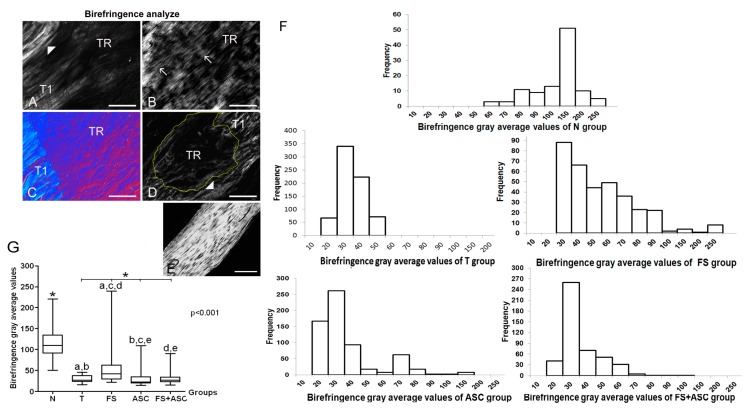
Images of birefringence of tendon longitudinal sections on 21st day using polarization microscopy (*n* = 5). The larger tendon axis was set 45° from the crossed polarizers. The variation of brightness intensity (gray levels) is due the variation of the collagen bundles organization. (**A**) T group: little birefringence brightness is observed in TR (tendon transected region) because of the disorganization of collagen bundles. T1 is the region which border the TR. (▶) remaining portion of the tendon located below the TR. (**B**) FS group: the increase in birefringence brightness was remarkable and a typical well-developed crimp (→) pattern was observed only in this group. (**C**) ASC group: image using DIC (differential interference contrast microscopy), where it is possible to visualize in red a smaller organization of the collagen bundles and in intense blue (according to Michel-Lévy’s table) the high degree of compaction of the collagen bundles. (**D**) FS + ASC group: observe a higher birefringence of the collagen fibers compared to the ASC group and observe an imbrication between collagen fibers that were not cut in T1 and fibrils in the TR (delimited by yellow line). (▶) remaining portion of the tendon located below the TR. (**E**) N group: collagen fibers exhibiting strong birefringence. Bar = 100 μm (**A**, **C**, **D**, **E**) and bar = 200 μm (**B**, **F**) Frequency histograms of birefringence gray average (GA) values expressed in pixels in the groups N, T, FS, ASC and FS + ASC, which reflect the variability of the collagen fibers organization on the TR region of the Achilles tendon. (**G**) Birefringence GA (pixels) median between the groups. The measurements data showed in graphics (f and g) were obtained with the larger tendon axis positioned at 45° from the crossed polarizers. (*) Significant differences between the group N and groups with transected tendons. The same letters between the groups correspond to a significant difference between them.

The analysis of tendons of different groups, under polarizing microscopy, showed higher birefringence values in FS in relation to all groups and in FS + ASC in comparison to the ASC group, indicating difference in the collagen bundles organization (Figure 8 A–G). N group exhibited higher birefringence values in relation to all transected groups. The image analysis of tendons sections from FS groups, showed a typical crimp arrangement in the transected region (Figure 8B). When DIC (Differential Interference Contrast) was used, which increases the contrast in unstained collagen bundles (for details see Vidal et al. [57]), it was possible to detect in the new ECM in formation, a blue color due to the presence of packed and organized collagen bundles and a background red color of unpacked or less organized collagen bundles (Figure 8C). The frequency histograms of birefringence gray average (GA) values showed the heterogeneous data distribution, with marked low birefringence values for group T and higher values especially for the FS group.

### 3.10. Biomechanical Properties of Tendons

**Figure 9:**
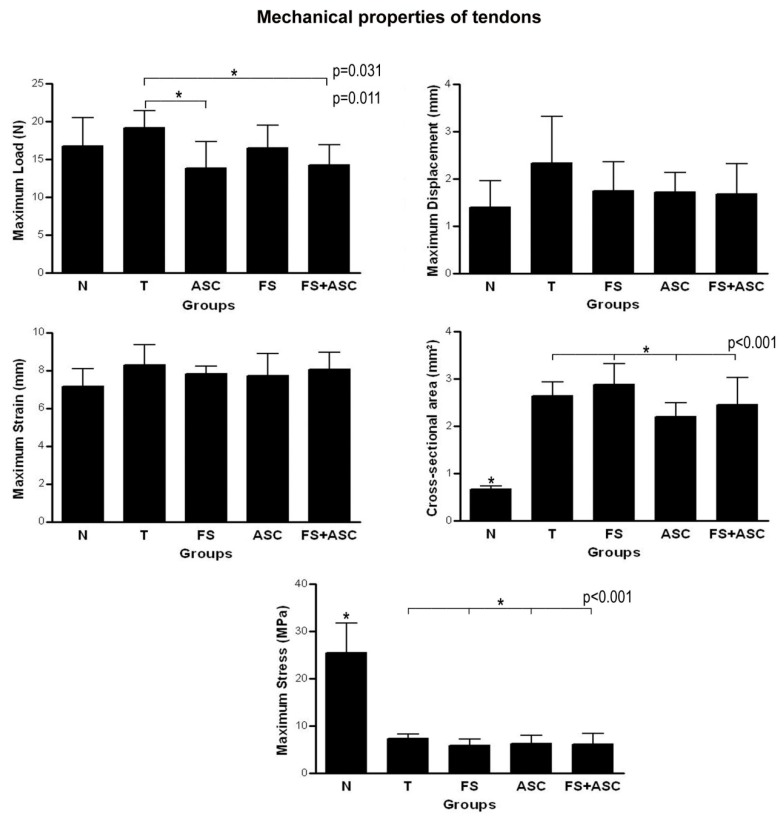
Mechanical properties of transected tendons from groups T, FS, ASC and FS + ASC (*n* = 8). (*) Significant difference between the groups.

The analysis of mechanical properties of transected tendons for the maximum load (N), maximum displacement (mm), maximum strain (mm), cross-sectional area (mm^2^) and maximum stress (MPa), showed some alterations (Figure 9). The T group had higher value of maximum load compared to the groups ASC (*p* = 0.011) and FS + ASC (*p* = 0.031). For the cross-sectional area and maximum stress, N group had lower and higher values, respectively, compared to the transected groups (*p* < 0.001).

For a systematic overview table of results see Table 3.

## 4. Discussion

In the present study, the 21st day after the transection was chosen because it marks the beginning of the remodeling phase, where there is a marked decrease in cellularity and formation of fibrous tissue [49,50,51], which is the focus of our study. In order to analyze the presence of ASC in isolation or associated with FS in the transected tendon region, in vivo imaging was used, where the striking result was observed in the ASC group due a high fluorescence intensity and area on the 1st day after injury, in relation to the FS + ASC group. Fibrin sealant works as a 3D scaffold that maintains cells grouped and possibly prevents the quantification of all labeled cells, while in its absence, cells are allowed to spread in the tissue. As a consequence of initial cell spreading (accommodation), increased labeling area was observed on the 1st day in the ASC group. But, as also demonstrated by Qiao et al. [62], our data showed a substantial ASC loss, when only stem cells are transplanted, from the 1st to the 2nd day. From the 2nd to the 7th day, no differences were observed among the groups when considering the fluorescence intensity and area. It is important to observe that only the FS + ASC group showed marking on the 14th day, with similar fluorescence intensity to that observed in previous periods and a larger area of fluorescence, possibly due to the fibrin sealant being degraded and consequently releasing the ASC. Our data supports the in vivo study by Wolbank et al. [63], which subcutaneously implanted in rats, fibrin clots with fluorophore-labeled and monitored their degradation for 21 days by in vivo imaging. The study showed that much of the fibrin clot was degraded by about 14 days and after 16 days, the degradation was total. Bensaïd et al. [64], through an in vitro study, demonstrated that fibrin scaffold together with human mesenchymal stem cells, depending on the concentration of fibrinogen and thrombin, is degraded within 14 to 15 days. Also in an agreement with our data, Spejo et al. [65] showed that the mesenchymal stem cells remain at region with 28 days post injury in glial scar region in the ventral funiculus of rats, evidencing that the sealant (the same as in the present study) kept these cells protected from the phagocytic system of the organism. This sealant is produced from the blood components of buffaloes, mainly fibrinogen, resulting in a fibrin clot and because it is heterologous, its fibrinolysis is delayed, causing it to act with an excellent scaffold for cells [29].

Orsi et al. [66] did not observe cytotoxicity of FS and thrombin-like enzyme (snake venom compound), both the same as in the present study, associated to mesenchymal stem cells. No marking was detected by in vivo imaging from the 15th day. However, the presence of a small number of ASC with CD90 and CD105 positive marking was showed into the newly formed matrix of the transected region in both ASC and FS + ASC groups on the 21st day, confirming the presence of ASC in the tendon. It is important to mention that the marking detected in tendons on the 21st day corresponds to a majority of live ASC since a few macrophages carrying PKH26 signal due to phagocytosis of dead ASC, were observed in the transected region of tendons.

The literature has already reported the paracrine effects of ASC on the modulation of cellular activity [67,68,69], as well as the effects of fibroblast secreted molecules on ASC activity [70]. In our injury model, the interaction between the transplanted ASC and the cells present in the transected region is evident since the ASC with CD90 e CD105 positive marking were identified in tendon ECM on the 21st day after transection. Some in vitro studies show the capacity of tenogenic differentiation of ASC in the presence of appropriate biological stimuli, such as growth factor, stress forces [71] and when cultivated in co-culture with tenocytes [70]. *Tnmd* is a good phenotype marker for tendon fibroblasts and acts as a regulator of cell proliferation and differentiation [72]. Our results showed significant increase of the *Tnmd* gene expression in the ASC group in relation to the groups without transplanted ASC, indicating a greater differentiation in tenocytes mainly of the transplanted ASC [70,73,74]. Thus, it is concluded that the molecular signaling present in the injury environment possibly affects the differentiation of ASC in tenocytes, only in the transected region of tendons. Considering the entire tendon, no differences were observed in the Tnmd amount, in a protein level, between the transected tendons. In addition to the *Tnmd*, Scx is also a key molecule involved in the process of tendon development. Scx is a transcription factor responsible for the differentiation of stem cells into tenocytes and is specifically detected in precursor populations of tendon cells [70,75]. Our results showed a trend to the increase of *Scx* expression in the ASC group in relation to the other transected groups, suggesting its relationship with the significant increase of the *Tnmd* expression. 

In order to analyze the effect of the treatments with ASC and FS reflecting in the entire tendon, collagen and tenomodulin, markers of tendon, were analyzed also in the entire tendons considering that the collagen fibers are also responsible for the mechanotransduction during gait, which is important for the cellular signaling in the transected region, affecting the recovery of the ECM at the site of injury. As well as it is important to consider that the molecular events in the transected region can also influence the organization and composition of the not damaged areas of tendons. In addition, collagen is the main constituent of tendons, mainly collagen type I, also being directly related to the recovery of the structure and biomechanical properties of tendons during repair process. Interestingly, the ASC group presented superior total collagen concentration, estimated by the hydroxyproline dosage, in relation to the N group. *Gdf5* and *Tgfb1* are related to the stimulation of collagen synthesis [26,70,76,77,78], supporting our results in which was observed a higher *Gdf5* expression in transected region of the ASC group, in comparison to N group and higher total collagen concentration in the entire tendon. However, no differences for the collagen I was observed between N and ASC groups. According to Uysal et al. [76], ASC applied on the injured calcaneus tendon of rabbits helped in tendon healing and increased the production of type I collagen.

Data from Peres [79] showed a marked increase of inflammatory infiltrate during the initial stage of healing of the skin of rats after an isolated application of either FS (the same FS used in our study) or after its association with ASC, in relation to the other sutured groups. The trend towards greater expression of *II1b* observed after the FS application in our study could be related to an inflammatory process with remnants still to the 21st day, considering the presence of fibrin until the 14th day in the injury region and period of its absorption [63]. Yücel et al. [80] showed that although the FS application increases inflammation on the first few days after application, the fibrin matrix helps tissue recovery.

The Yücel et al. [80] study mentioned above supports our structural data. Higher values of birefringence were detected in the FS group in comparison to all groups, followed by the FS+ASC and ASC groups in relation to T, respectively, reflecting greater organization of the collagen bundles. Considering that tissue reorganization after injury is extremely important for the recovery of the tendon functionality, our structural result is highly relevant considering the 21st day of repair, because depending on the extent of the injury, the tendon may take months for complete healing [81]. The use of FS acted as a scaffold for resident tendon cells, as well as for the interaction of ASC transplanted with resident cells of the transected region since both FS and FS + ASC groups had higher birefringence values, showing that fibrin is slowly replaced by connective tissue. A study of Tuan et al. [82] supports our structural results because demonstrated that the cultured fibroblasts together with the fibrin gel, synthesize collagen and actively reorganize the fibrin matrix which is largely replaced by collagen fibrils. Regarding the analysis of genes involved in ECM remodeling, no striking results were observed in relation to the expression of *Lox*, *Dcn*, *Mmp2*, *Mmp9* and *Timp2* in the transected tendons, suggesting the involvement of other genes in the higher collagen fibers organization observed specially in the FS, followed by FS + ASC and ASC groups

In the biomechanical analysis, no differences were observed between the transected groups for the stress parameter on the 21st day, supporting no differences in the amount of collagen type I in the entire tendon, which is directly related to the resistance of tendon. Although, the literature points to the beneficial effects of FS and ASC on the biomechanics of tendons in the healing process [83]. Therefore, it is possible to conclude that the structural data did not directly reflect the biomechanics of the tendons, perhaps due to the recovery time, 21 days, which is marked by the beginning of the remodeling process. A point to be considered is that the number of crosslinks present in tendons treated with ASC and with FS isolated or in combination, reflected in the higher organization of these tendons but was not enough to warrant a more resistant tendon on the 21st day after transection. We believe that long-term time point analysis could demonstrate that transected tendon treated with FS exhibited superior strength in comparison with T group.

According to our data, transplanted ASC migrated to the transected region and consequently the ASC up-regulated the *Tnmd* expression suggesting a differentiation of transplanted cells in tenocytes and the ASC increased the collagen fibers organization. The application of FS alone was able to improve the molecular organization of the collagen fibers but when associated with the ASC, a higher number of cells was kept in the transected region, with the ASC protected from the phagocytic system. In conclusion, our data suggest this FS to be a good scaffold for treatment during tendon repair because it was the most effective one regarding tendon organization recovering, followed by the FS treatment associated with ASC and finally by the transplanted ASC on the 21st day. Further investigations in long-term time points of the tendon repair are needed to analyze if the higher tissue organization found with the FS scaffold will improve the biomechanics of the tendons.

## Figures and Tables

**Table 1:** Hydroxyproline concentration (mg/g tissue) in the entire tendon (n = 8). (*) Significant difference between N and ASC groups.

Groups	N	T	FS	ASC	FS+ASC
Hydroxyproline (mg/g tissue)	100.8 ± 7.5 *	103.6 ± 9.1	104.9 ± 8.5	107.9 ± 6.5 *	101.6 ± 6.7

* *p* < 0.041.

**Table 2:** Total number of fibroblasts: no differences were observed between the transected tendons. (*) Significant difference between the N group and the transected groups (*n* = 3).

Groups	N	T	FS	ASC	FS + ASC
Fibroblasts	26.6 ± 3.8 *	88.7 ± 11.7 *	93.9 ± 9.8 *	96.7 ± 12.3 *	90.5 ± 14.2 *

* *p* < 0.001.

**Table 3:** Systematic overview table of results.

	N	T	FS	ASC	FS + ASC
In vivo imaging for ASC detection on tendon	-	-	-	Detection of labeled-ASC until the 7th day	Detection of labeled-ASC until the 14th day
Cell migration assay	-	-	-	Presence of ASC on the 21st day	Presence of ASC on the 21st day
Macrophages identification	Presence of few macrophages	Presence of few macrophages	Presence of few macrophages	Presence of few macrophages carring PKH26 signal	Presence of few macrophages carring PKH26 signal
Genes expression analysis	Lower expression of *Mmp2*, *Mmp9*, *Timp2*, *Gdf5*, *Scx* and *Tnmd*; and higher expression of *Tnf* and *Dcn* in relation to T, FS, ASC and FS + ASC groups	Lower *Tnmd* expression in relation to ASC group	Lower *Tnmd* expression in relation to ASC group	Higher *Tnmd* expression in relation to T and FS groups	No differences between the treatments
Total collagen concentration (entire tendon)	Lower concentration in relation to ASC group	No differences between the treatments and N group	No differences between the treatments and N group	No differences between the treatments and higher concentration in relation to N group	No differences between the treatments and N group
Collagen I and Tnmd quantification (entire tendon)	Lower amount in relation to T, FS and FS+ASC groups	No differences between the treatments	No differences between the treatments	No differences between the treatments and N group	No differences between the treatments
Total number of fibroblasts in the TR	Higher number of cells in relation to T, FS, ASC and FS+ASC groups	No differences between the treatments	No differences between the treatments	No differences between the treatments	No differences between the treatments
Collagen fibers organization measurements	Higher birefringence in relation to T, FS, ASC and FS+ASC groups	Lower birefringence in relation to FS, ASC and FS+ASC groups	Higher birefringence in relation to T, ASC and FS+ASC groups	Higher birefringence in relation to T group	Higher birefringence in relation to T and ASC groups
Biomechanical properties of tendons	*Maximum Load:* no differences in relation to T, FS, ASC and FS+ASC groups; *Cross-sectional area:* lower value in relation to T, FS, ASC and FS+ASC groups; *Maximum Displacement and Strain:* no differences in relation to T, FS, ASC and FS+ASC groups; *Maximum Stress:* higher value in relation to T, FS, ASC and FS+ASC groups	*Maximum Load:* higher value in relation to ASC and FS+ASC groups; *Cross-sectional area:* no differences between the treatments; *Maximum Displacement and Strain:* no differences between the treatments; *Maximum Stress:* no differences between the treatments	*Maximum Load:* no differences between the treatments; *Cross-sectional area:* no differences between the treatments; *Maximum Displacement and Strain:* no differences between the treatments; *Maximum Stress:* no differences between the treatments	*Maximum Load: lower value in relation to T group; Cross-sectional area:* no differences between the treatments; *Maximum Displacement and Strain:* no differences between the treatments; *Maximum Stress:* no differences between the treatments	*Maximum Load: lower value in relation to T group; Cross-sectional area:* no differences between the treatments; *Maximum Displacement and Strain:* no differences between the treatments; *Maximum Stress:* no differences between the treatments
